# Protective effects of butorphanol in oleic acid-endotoxin “two-hit” induced rat lung injury by suppression of inflammation and apoptosis

**DOI:** 10.1038/s41598-024-53483-5

**Published:** 2024-06-20

**Authors:** Yanlei Zheng, Ronghua Hu, Jinrong Hu, Lina Feng, Shi Li

**Affiliations:** Department of Intensive Care Medicine, Hubei Cancer Hospital, Tongji Medical College, Huazhong University of Science and Technology, Wuhan, 430079 China

**Keywords:** Drug discovery, Medical research, Molecular medicine

## Abstract

Butorphanol is widely used as an anesthetic drug, whether butorphanol could reduce organ injury and protecting lung tissue is unknown. This study explored the effects of butorphanol on ALI and investigated its underlying mechanisms. We established a “two-hit” rat model and “two-hit” cell model to prove our hypothesis. Rats were divided into four groups [control, “two-hit” (OA + LPS), “two-hit” + butorphanol (4 mg/kg and 8 mg/kg) (OA + LPS + B1 and OA + LPS + B2)]. RPMVE cells were divided into four groups [control, “two-hit” (OA + LPS), “two-hit” + butorphanol (4 μM and 8 μM) (OA + LPS + 4 μM and OA + LPS + 8 μM)]. Inflammatory injury was assessed by the histopathology and W/D ratio, inflammatory cytokines, and arterial blood gas analysis. Apoptosis was assessed by Western blotting and flow cytometry. The effect of NF-κB p65 was detected by ELISA. Butorphanol could relieve the “two-hit” induced lung injury, the expression of TNF, IL-1β, IL-6, and improve lung ventilation. In addition, butorphanol decreased Bax and cleaved caspase-3, increased an antiapoptotic protein (Bcl-2), and inhibited the “two-hit” cell apoptosis ratio. Moreover, butorphanol suppressed NF-κB p65 activity in rat lung injury. Our research showed that butorphanol may attenuate “two-hit”-induced lung injury by regulating the activity of NF-κB p65, which may supply more evidence for ALI treatment.

## Introduction

Sepsis is clinically deemed fatal systemic aberrant inflammatory responses, and severe bloodstream infection causes coagulation dysfunction and immune suppression that can rapidly become life-threatening^1–3^. Excessive macrophage activation releases massive amounts of cytokines and inflammatory factors that accelerate sepsis-induced acute lung injury (ALI)^4^. ALI in its most severe form and acute respiratory distress syndrome (ARDS), are characterized by extensive inflammatory factors in the lungs that damage the lung epithelium and endothelium, which cause pulmonary vascular permeability and the deterioration of gas exchange^5^.

Current research shows that the pathogenesis of ARDS caused by primary protopathy still exists in surgical trauma, intestinal bacterial toxins to the blood and other “two-hit” factors^6,7^. The theoretical basis of “two-hit” is the first-hit (severe injury or infection, etc.) causing systemic inflammatory response syndrome (SIRS), two-hit (posttraumatic infection, inappropriate resuscitation, etc.) On this basis, they suffer and trigger the loss of control of SIRS, inflammatory reactions, and anti-inflammatory reaction imbalance, leading to ARDS and even multiple organ dysfunction syndrome (MODS)^7,8^. Thus, the “two-hit” model type can better simulate the human ARDS process, establishing an ideal “two-hit” animal model of ARDS revealing the pathogenesis of the ARDS mechanism^7,8^. Current ARDS model types include lung lavage type, salt acid suction type, oleic acid type, endotoxin type, etc^9^. In this study, endotoxin-oleic acid was used to construct a “two-hit” ARDS model to understand the pathogenesis of ARDS.

**Figure 1:**
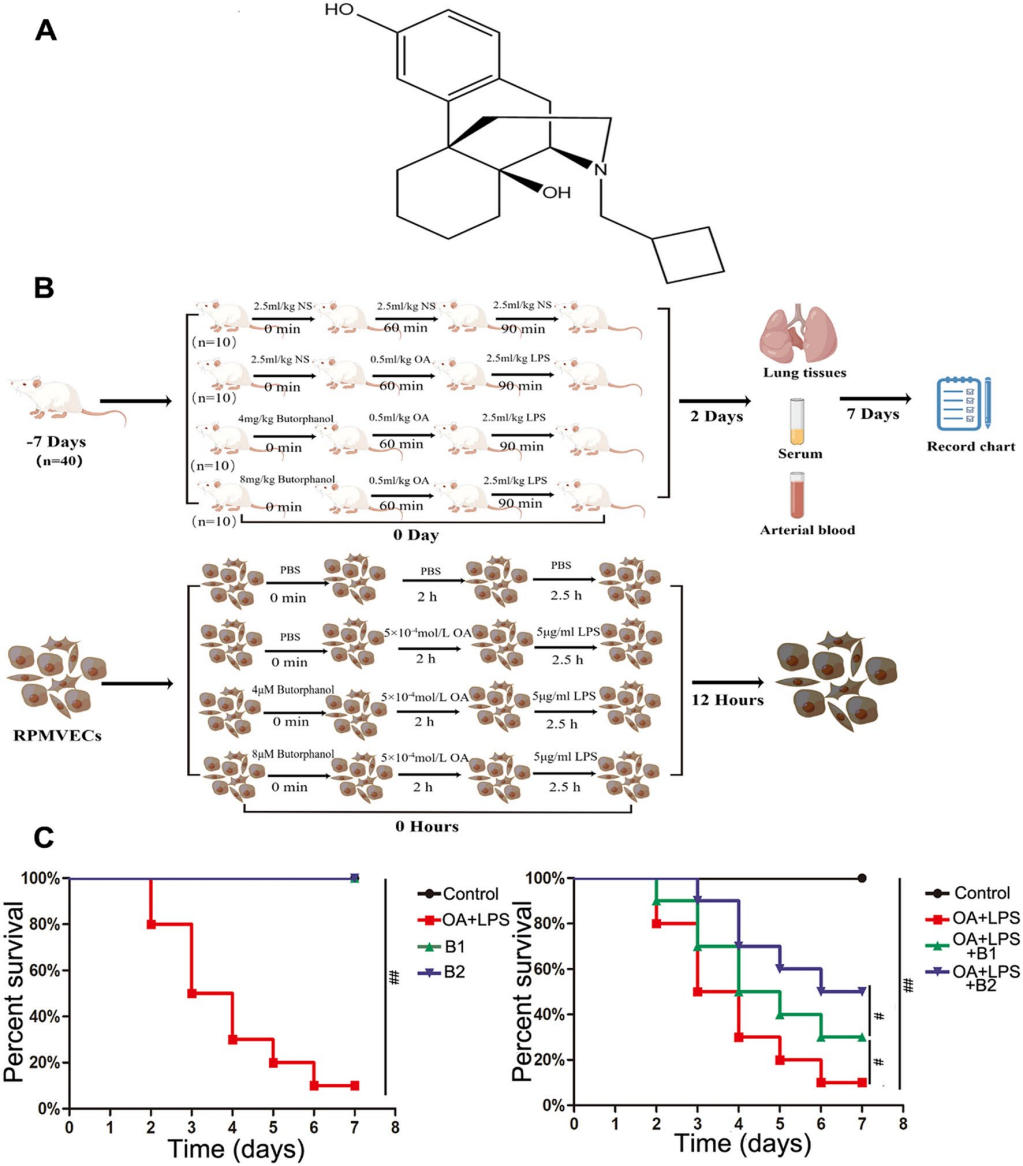
The chemical structure of butorphanol and effects of butorphanol in “two-hit” rat ALI on the survival ratio. (**A**) The chemical structure of butorphanol; (**B**) Schematic diagram of the trial (Figdraw); (**C**) Percent survival of control and B-treated rats with lung injury was observed for 7 days. Values are means ± SEMs, n = 10. (^##^*p* < 0.01, ^#^*p* < 0.05).

Butorphanol (chemical structure is shown in Fig. 1A) is a new opioid receptor anesthetic drug that is a strong κ-receptor agonist and a weak μ-receptor agonist-antagonist^10^. Butorphanol is widely applied in the clinic as an analgesic, while many studies have established the protective effect of κ-receptors on the heart, lung, brain, and other important organs^11–13^. Huang et al.^11^ reported that butorphanol attenuates myocardial ischemia reperfusion injury by inhibiting mitochondria–mediated apoptosis in mice. Huang et al.^12^ butorphanol reduces the neuronal inflammatory response and apoptosis. Luan et al.^13^ shown that butorphanol promotes macrophage phenotypic transition to inhibit inflammatory lung injury. Some reports have shown that nuclear factor kappa-B (NF-κB) plays a key role in regulating inflammation and apoptosis through a variety of signaling pathways, such as TLR4/NF-κB, NF-κB/NLRP3 signaling^14,15^. However, whether butorphanol can regulate the mechanism of sepsis-induced ALI via NF-κB requires further research.

## Results

### Butorphanol pretreatment decreases mortality in “two-hit” rat lung injury

To confirm the effect of butorphanol against “two-hit”-induced acute lung injury, we monitored rat survival in the control and treatment groups for 7 days (Fig. 1C). The rats with “two-hit” induced lung injury presented a 7-day survival rate of 10%, and all control rats treated with or without butorphanol survived. Butorphanol pretreatment (OA + LPS + B2) increased the 7-day survival rate to *50% (*^*#*^*p* < *0.05 vs.* OA + LPS) and the survival rate to 30% in the OA + LPS + B1 group (^*#*^*p* < *0.05 vs.* OA + LPS + B2).

### Butorphanol alleviated “two-hit” induced inflammation injury and apoptosis in vivo

**Figure 2:**
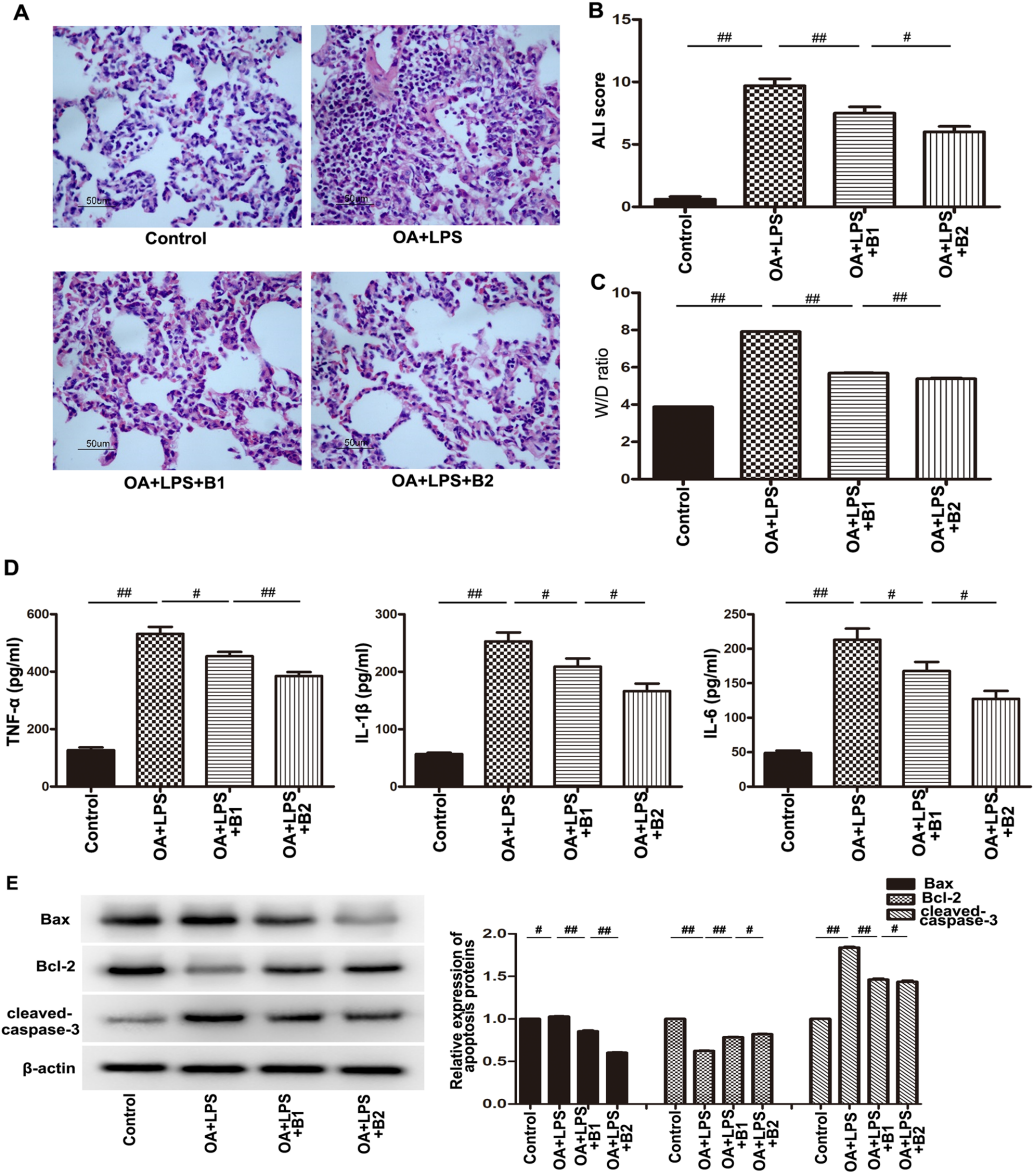
Effect of butorphanol on rat acute lung injury in vivo. (**A**) The morphological change was analyzed among these groups. (**B, C**) The ALI score was assessed by HE staining and the W/D ratio was detected in rats. (**D**) The proinflammatory cytokine expression levels of TNF-α, IL-1β and IL-6 were calculated by ELISA. Values are means ± SEMs, n = 10, ^(##^*p* < 0.01,^#^*p* < 0.05*)*. (**E**) The apoptosis proteins Bax, Bcl-2 and cleaved caspase-3 were estimated by western blotting in “two-hit”-induced ALI rats. β-Actin was used as an internal control. All data are displayed as the ratio of Bax/β-actin, Bcl-2/β-actin and cleaved caspase-3/β-actin. The experiment was repeated three times, and representative results are shown. (^##^*p* < 0.01, ^#^*p*<0.05).

We established a “two-hit” rat model to show the protective effect of butorphanol against ALI in vivo. Rat lung histological examination through HE staining was assessed to determine the degree of damage to lung tissue structure. There was significant tissue damage and destruction that included alveolar structural failure, edema, hyperemia, and a mass of inflammatory cell infiltration compared with the control group (Fig. 2A). However, histologic analysis showed that butorphanol (4 mg/kg and 8 mg/kg) attenuated “two-hit”-induced lung tissue destruction. Additionally, the ALI score and lung wet/dry weight ratio were also used to evaluate the degree of lung tissue injury (Fig. 2B, C). The analysis showed that the ALI score of 9.7 and the W/D ratio of 7.9 in the OA + LPS group were higher than those in the butorphanol groups (^*##*^*p* < *0.01*) and that the degree of lung injury was related to the concentration (^*#*^*p* < *0.05, *^*##*^*p* < *0.01*).

The expression levels of proinflammatory cytokines (TNF-α, IL-1β and IL-6) in serum were confirmed by ELISA. The expression levels of cytokines in the OA + LPS group were markedly increased compared with those in the control group (^*##*^*p* < 0.01). The expression levels of these cytokines after butorphanol injection were decreased compared with those in the OA + LPS group and exhibited concentration dependence (^*##*^*p* < *0.01* or ^*#*^*p* < *0.05*) (Fig. 2D).

In the blood gas analysis, in the OA + LPS group, the pH value and PO2 were significantly decreased compared with those in the control group (^*a*^*p* < *0.01*), and the PCO2 level was increased compared with that in the control group (^a^*p* < 0.01). Furthermore, in the different doses of butorphanol treatment group, the results of the PH value and PO2 were increased and the PCO2 level was lower compared with the OA + LPS group (^b^*p* < 0.05). However, no differences in the pH value and PCO2 were observed between the two butorphanol groups (^c^*p* > 0.05, ^d^*p<0.05*) (Table 1).

**Table 1:** Arterial blood gas in the rats.

	Control	OA + LPS	OA + LPS + B1	OA + LPS + B2
PH	7.39 ± 0.06	7.14 ± 0.78^a^	7.21 ± 0.07^ab^	7.25 ± 0.08^abc^
PO_2_	110.17 ± 12.44	62.17 ± 7.19^a^	74.08 ± 8.26^ab^	80.92 ± 7.47^abd^
PCO_2_	34.75 ± 6.26	57.25 ± 8.06^a^	49.17 ± 8.52^ab^	47.08 ± 7.26^abc^

*PH* pH value; *PO2* partial pressure of oxygen, *PCO2* partial pressure of carbon dioxide,

^a^p < 0.05 vs. Control, ^b^p < 0.05 vs. OA + LPS, ^c^p > 0.05 vs. OA + LPS + B1, ^d^p < 0.05 vs. OA + LPS + B2.

To evaluate the effect of butorphanol on “two-hit” induced apoptosis, the protein expression level of proapoptotic proteins (Bax, cleaved caspase-3) and anti-apoptotic protein (Bcl-2) was assessed by western blotting. As shown in Fig. 2E, the butorphanol groups had decreased expression levels of Bax and cleaved caspase-3, while the expression level of Bcl-2 increased compared with the OA + LPS group (^##^*p* < *0.01*).

### Butorphanol alleviated oleic acid-endotoxin “two-hit” induced the expression of proinflammation cytokines and apoptosis in vitro

**Figure 3:**
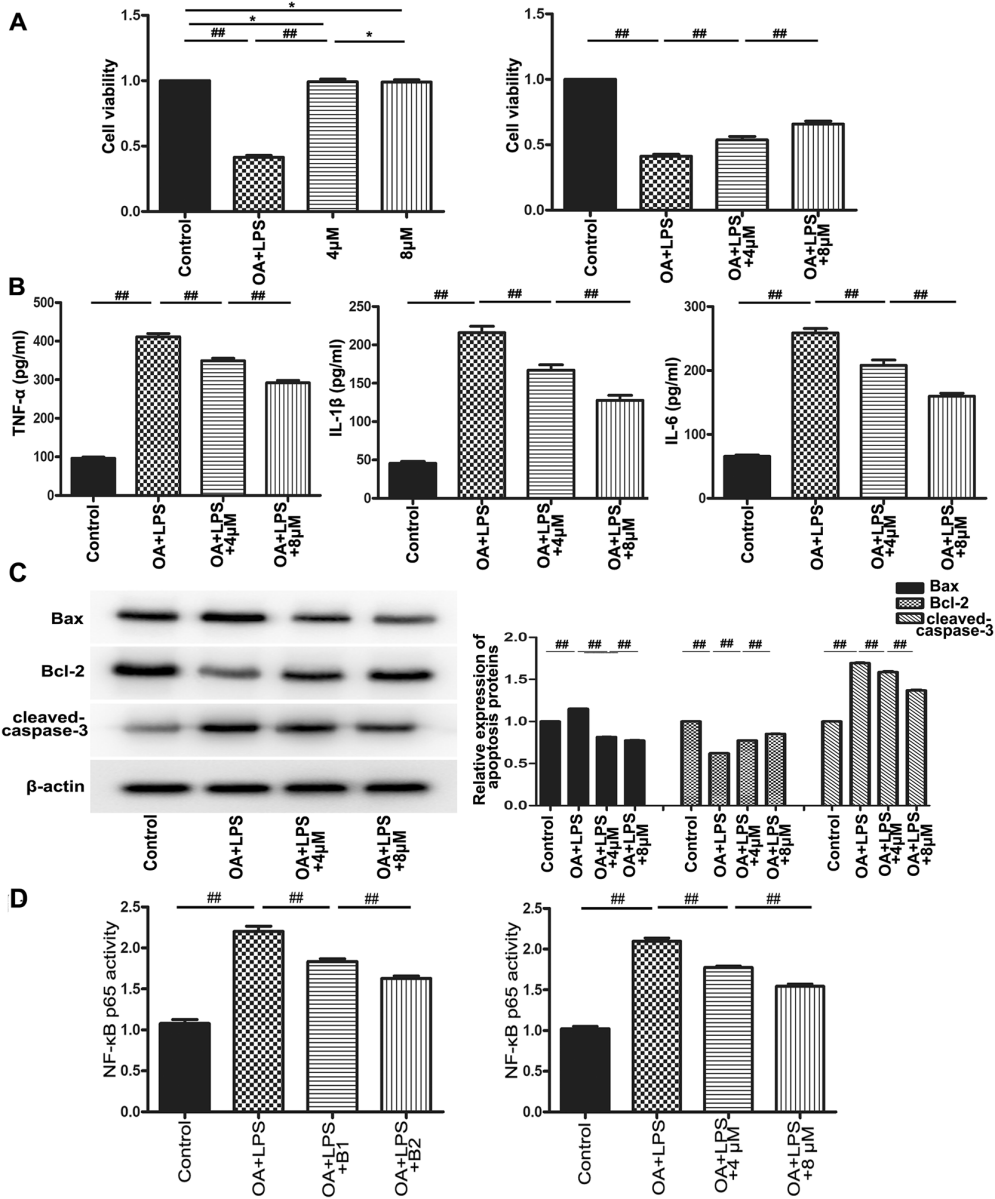
Effect of butorphanol on “two-hit” injury in vitro and the effect of butorphanol on NF-κB p65 activity in “two-hit” injury in vivo and in vitro. (**A**) RPMVECs viability was assessed by CCK-8 assay, n = 10. (**B**) The proinflammatory cytokine expression levels of TNF-α, IL-1β and IL-6 were calculated in vitro by ELISA, n = 8. All data are presented as the means ± SEMs, ^##^*p* < 0.01, ^*^p > 0.05. (**C**) The apoptosis proteins Bax, Bcl-2 and cleaved caspase-3 were detected by western blotting. β-Actin was used as an internal control. All data are displayed as the ratio of Bax/β-actin, Bcl-2/β-actin and cleaved caspase-3/β-actin. The experiment was repeated three times, and representative results are shown. ^##^*p* < 0.01*. (D*) The activation of NF-κB p65 was detected by ELISA in vivo and in vitro, n = 6. All data are presented as the means ± SEMs, ^##^*p* < 0.01.

To further assess the effect of butorphanol, we established a “two-hit” model to simulate the development of lung injury in vitro. The viability of RPMVECs was detected by CCK-8 assay. The results indicated that the viability of RPMVECs was not significantly altered except in the OA + LPS treatment group, which showed that butorphanol had no effect on the viability of normal lung microvascular endothelial cells (^*^p > *0.05, *^*##*^*p* < *0.01).* Moreover, the cell viability of the butorphanol treatment groups was increased compared with that of the OA + LPS group (^*##*^*p* < *0.01*) (Fig. 3A).

The expression levels of proinflammatory cytokines (TNF-α, IL-1β and IL-6) in RPMVECs were evaluated by ELISA. The expression levels of cytokines in the OA + LPS group were markedly increased compared with those in the control group (^*##*^*p* < 0.01). The expression levels of these cytokines after butorphanol treatment were lower than those in the OA + LPS group and exhibited concentration dependence (^*##*^*p* < *0.01*) (Fig. 3B).

To clarify whether butorphanol reduced RPMVECs apoptosis, apoptosis proteins were detected. The results showed that butorphanol decreased the protein expression levels of Bax and cleaved caspase-3, and the expression level of Bcl-2 increased compared with that in the OA + LPS group (^##^*p* < *0.01*) (Fig. 3C).

As shown in Fig. 3D, in vivo and in vitro experiments showed that the activity of NF-κB p65 was enhanced in the “two-hit” group compared with the control group (^*##*^*p* < *0.01).* Treatment with different doses of butorphanol significantly inhibited the activity of NF-κB p65 (^*##*^*p* < *0.01).* It has been shown that butorphanol may inhibit the “two-hit”-induced activity of NF-κB p65 in a concentration-dependent manner.

### Butorphanol alleviated the apoptosis ratio in “two-hit”-induced RPMVECs

**Figure 4:**
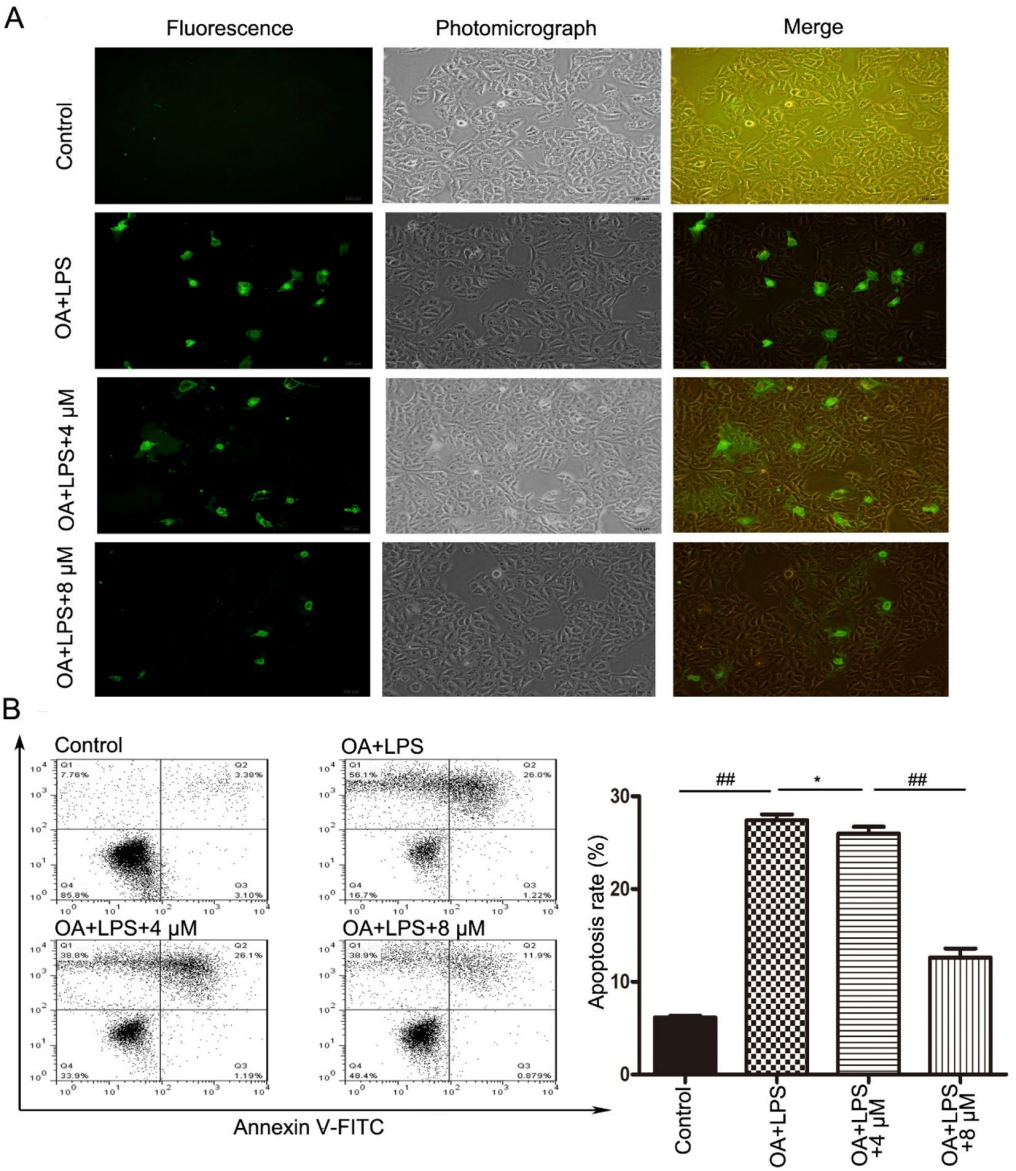
Butorphanol alleviated the “two-hit” inducing RPMVECs apoptosis. (**A**) The apoptotic cells emitted light-red and light-green fluorescence under fluorescence microscopy and showed a change in morphology under electron microscopy. (**B**) The apoptosis ratio in RPMVECs was detected by flow cytometry. Values are means ± SEMs, n = 3. ^##^*p* < 0.01, ^*^p > 0.05

To further confirm the protective effect of butorphanol, fluorescence microscopy was used to show the change in morphology and detachment from neighboring cells. In view, the apoptotic cells emitted light-red and light-green fluorescence by PI staining. Compared with the control group, the treatment groups emitted red‒green fluorescence, and the butorphanol groups exhibited a decreased quantity of stained cells (Fig. 4A).

Apoptosis in RPMVECs was measured by the Annexin V-FITC/PI staining method to estimate the protective effect of butorphanol. Compared with the control group, the treatment groups displayed markedly increased cell mortality and apoptosis rates (^##^*p* < *0.01)*. However, the apoptosis rate of butorphanol group(4 μM) has no significant difference compared with the OA + LPS group (27.41 ± 1.07%*vs*.25.76 ± 1.59%, ^*^p > *0.05*), but the mortality rate existed significant difference (52.03 ± 3.05%*vs*.36.64 ± 1.78%, ^##^*p* < *0.01*). Meanwhile, the apoptosis rate of two different doses of butorphanol was markedly different (25.76 ± 1.59% *vs*. 12.62 ± 1.69%, ^##^*p* < *0.01*) (Fig. 4B).

## Discussion

Our current study confirmed the hypothesis that butorphanol could alleviate “two-hit”-induced ALI and participate in apoptosis progression. Our research mainly presents several aspects: (1) In an in vivo experiment, butorphanol inhibited lung tissue damage, alveolar structural failure, and inflammatory cell infiltration, as shown by HE staining. (2) The ALI score, W/D ratio of lung tissue and indicators of arterial blood gas were used to assess the severity of “two-hit”-induced ALI, and the protective effect of butorphanol was related to its concentration. (3) In the in vivo and in vitro experiments, our study also showed that butorphanol suppressed the release of proinflammatory cytokines such as TNF-α, IL-1β and IL-6. (4) Butorphanol inhibited the expression of the proapoptotic factors Bax and cleaved caspase-3 and the expression of the antiapoptotic factor Bcl-2 in vivo and in vitro. (5) In our studies, butorphanol inhibited the activity of NF-κB p65 in a concentration-dependent manner in vivo and in vitro. (6) The anti-apoptotic effect of butorphanol was also estimated by immunofluorescence analysis and flow cytometry methods in vitro. In addition, butorphanol markedly reduced RPMVECs death.

Butorphanol is widely used in the clinic as a lipid-soluble anesthetic drug with efficient analgesic effects^12,16,17^^.^ In recent years, a series of reports have shown that butorphanol decreases the levels of pro-inflammatory cytokines or increases the levels of anti-inflammatory cytokines to reduce organ injury and cell apoptosis^11–13,18^. However, the underlying anti-inflammatory mechanism of butorphanol has not been fully demonstrated. Clinically, the occurrence and development of ARDS is usually caused by multiple pathogenic factors, but the ALI animal model usually focuses on a singular cause in trials. To better simulate the progression of ALI in the clinic, oleic acid and/or endotoxin were used to construct a “two-hit” ALI model in our trial.

Our animal trial demonstrated that oleic acid (0.5 ml/kg), as the initial injury cause, was previously infected and induced secondary lung injury with LPS (2.5 mg/kg), leading to enhanced ALI compared with a single exposure factor^8,9^. These treatment group rats showed clinical symptoms of tachypnea, distress and reduced activity compared with the untreated group. In the survival experiment, the rats with “two-hit”-induced lung injury presented a 7-day survival rate of 10%, but all control and single butorphanol-treated groups survived. Butorphanol pretreatment (OA + LPS + B2) increased the 7-day survival rate to 50% compared with the “two-hit” group (^*#*^*p* < *0.05*), showing the protective effect of butorphanol against ALI. Histological changes in lung tissue revealed edema, hyperemia, inflammatory cell infiltration and extensive alveolar wall thickness among the treatment groups. Meanwhile, we further observed that the protective effect of butorphanol could relieve the edema and destruction of pulmonary vascular endothelial cells by the W/D ratio and ALI score, and a higher concentration of butorphanol (8 mg/kg) had a stronger protective effect than a lower concentration (4 mg/kg). We further observed that the expression of proinflammatory cytokines (TNF-α, IL-1β and IL-6) was increased in the “two-hit” groups, butorphanol suppressed the expression of these cytokines, and the protective effect was positively correlated with concentration in vivo and in vitro. (^##^*p* < *0.01 or* ^#^*p* < *0.05*). The results were in accordance with these previous reports that butorphanol reduced brain and neurological damage and relieved the expression of inflammatory cytokines^12,19^. Arterial blood gas analysis was also one of the important criteria to assess the gas exchange extent and destruction of the alveolar wall^20,21^. The results showed that butorphanol increased arterial blood PO2 and pH values, and PCO2 decreased compared with the OA + LPS group. However, the different doses of butorphanol had no significant difference between the pH value and PCO2 in the in vivo experiment.

Recent evidence indicated that butorphanol could alleviate the elevation of pro-apoptotic genes and may suppress apoptosis^12^. We estimated the expression levels of proapoptotic proteins (Bax and cleaved caspase-3) and an antiapoptotic protein (Bcl-2) by western blotting. The results showed that butorphanol downregulated the levels of the proapoptotic proteins Bax and cleaved caspase-3 and upregulated the level of the antiapoptotic protein Bcl-2 in vivo and in vitro (^#*#*^*p* < *0.01*).

Many previous reports^14,22,23^have indicated that the inflammatory response and the expression of proinflammatory cytokines, such as TNF-α and IL-1β, are relieved by inhibiting the NF-κB signaling pathway and are involved in the regulation of cell apoptosis. Previous reports^24^ have shown that TNF-α indirectly leads to apoptosis by increasing the activities of caspase3 and caspase8. Furthermore, the pro-apoptotic genes Bax and Bad, the anti-apoptotic genes Bcl-2 and Bcl-xL interacting killer and p65 transportation to the nucleus also participate in the process of apoptosis^12,24^. We calculated the activity of NF-κB p65 in vivo and in vitro, and the results showed that butorphanol inhibited the “two-hit”-induced activity of NF-κB p65 (^#*#*^*p* < *0.01*). The data showed that butorphanol inhibited the activity of NF-κB p65, reduced the levels of TNF-α and then led to the development of apoptosis. We further confirmed that butorphanol reduced the number of apoptotic cells emitting light-green and light-red fluorescence by fluorescence microscopy. Analysis of the RPMVECs apoptosis rate indicated that butorphanol treatment decreased the early and late apoptosis rates compared with the OA + LPS group. This further demonstrated the protective effect of butorphanol on “two-hit” lung injury. Weiqing Tang^25^ et al. also found that butorphanol alleviates lipopolysaccharide-induced inflammation and apoptosis of cardiomyocytes by upregulating KOR expression. Previous studies also revealed^13^ the protective effect of butorphanol on cell viability and its inhibitory effects on the inflammatory response and apoptosis by inhibiting other signaling pathways, such as the p38/JNK/ATF2/p53 pathways. In summary, our present in vivo and in vitro study provided evidence that butorphanol attenuates “two-hit”-induced lung injury and apoptosis and may be involved in this process by regulating the activity of NF-κB p65, which may supply more evidence for the therapeutic effects of butorphanol in addition to participating in anesthesia and the underlying mechanism of sepsis treatment.

## Methods

### Main reagents

Butorphanol was purchased from Hengrui (Jiangsu, China). Lipopolysaccharide was obtained from Sigma (St. Louis, USA). Oleic acid was purchased from XiLong Scientific (China). TNF-α, IL-1β, and IL-6 ELISA kits were obtained from Abcam (UK). The NF-κB p65 transcription factor assay kits were obtained from Cayman Chemical. Antibodies against Bax, Bcl-2 and cleaved caspase-3 were obtained from Cell Signaling Technology, Inc. (Danvers, MA, USA). β-actin antibody, HRP-conjugated anti-rabbit and anti-mouse were purchased from Santa Cruz Biotechnology, Inc. (Dallas, TX, USA). The Annexin V-fluorescein isothiocyanate (FITC) kit was acquired from BestBio Co. (Shanghai, China).

### Rat “two-hit” modeling and grouping

A total of 40 Sprague‒Dawley (SD) male rats (200–220 g body weight, 6–8 weeks old) purchased from Tongji Medical College, Huazhong University of Science and Technology Animal Center (Wuhan China). Rats were maintained in a stable temperature of 22–24 °C, 60% humidity, under a 12-h dark/light cycle with free access to food and water for 7 days. All the rats were injected by the tail vein, the day of the first injection was considered as day 0 and observed for a week. Rats were randomly divided into four groups: control group [2.5 ml/kg normal saline (NS) i.v. given at 0 min, 60 min and 90 min); OA + LPS group [2.5 ml/kg NS i.v. given at 0 min, 0.5 ml/kg OA i.v. given at 60 min, and 2.5 mg/kg LPS, i.v. administered at 90 min]; B1 group and B2 group [4 mg/kg or 8 mg/kg butorphanol i.v. given at 0 min, 0.5 ml/kg OA i.v. given at 60 min, and 2.5 mg/kg LPS, i.v. given at 90 min] (Fig. 1B). For the research, all rats were housed at a constant temperature of 23–25 °C and monitored for 48 h^8,26^. All the rats’ activity, appetite, breathing and heart rate were recorded daily for a week, and rats’ death was judged by heartbeat, breathing and body temperature.

### Rat pulmonary microvascular endothelial cells (RPMVECs) culture and “two-hit” modeling

RPMVECs were purchased from iCell Bioscience Inc, Shanghai. Cells were cultured in DMEM (Gibco, Fisher Scientific, Inc.) with 10% FBS, 1.5 g/l glucose and 1% penicillin‒streptomycin at 37 °C in a humidified atmosphere containing 5% CO2. Cells were divided into the following four groups: control group [PBS given at 0 min, 2 h and 2.5 h]; OA + LPS group [PBS given at 0 min, 5 × 10^-4^ mol/L OA given at 2 h, and 5 μg/ml LPS given at 2.5 h]; 4 μM group and8μM group[4 μM,8 μM Butorphanol given at 0 min and 5 × 10^-4^ mol/L OA given at 2 h and 5 μg/ml LPS given at 2.5 h] (Fig. 1B); The cells were used for further experiment after 12 h cultivation at 37 ℃^12,27^.

### CCK-8 cell viability assay

RPMVECs viability was detected by the Cell Counting Kit-8 (MedChem Express, New Jersey, USA) following the manufacturer’s guidelines. The absorbance was measured at a wavelength of 450 nm by an automatic porous spectrophotometer (Molecular Devices, USA).

### Histological examination

The upper lobe of the right lung was isolated, perfused, and fixed with 4% formaldehyde, embedded and cut into 5-μm sections. The samples were stained with hematoxylin and eosin (HE) for the analysis of lung injury histopathological changes and assessment of injury score. A scoring system was used to assess the degree of lung injury, and the criteria were as follows: 0: normal; 1: mild pulmonary interstitial hyperemia and leukocyte infiltration; 2: alveolar edema and moderate lung structural damage; 3: moderate alveolar structure collapse and massive inflammatory cell infiltration; and 4: severe alveolar structure destruction and substantial leukocyte infiltration^28^.

### Immunofluorescence analysis and flow cytometry

RPMVECs were digested with trypsin and resuspended to 5 × 10^7^–1 × 10^8^/L in each group. These cells were centrifuged at 500 r/min for 5 min at 4 °C, washed three times with cold PBS and resuspended in 400 μl binding buffer. Annexin V-FITC staining solution (5 μl) was added and incubated at room temperature for 15 min in darkness, and then 10 μl propidium iodide was added and incubated at room temperature without light for 5 min. Apoptosis was observed using fluorescence microscopy and analyzed by flow cytometry. The results were processed by CellQuest software version 3.3 (BD Biosciences, San Jose, CA, USA).

### Detection of ALI inflammatory factors, activity of NF-κB and arterial blood gas analysis

The levels of cytokines (TNF-α, IL-1β and IL-6) and activity of NF-κB in serum and RPMVECs were measured according to ELISA kits (R&D Systems, Inc., USA; NF-κB p65 Transcription Factor Assay Kit) following the manufacturer’s instructions. The rat common carotid arterial blood samples were collected 0.4 ml into an ABL700 Radiometer (Radiometer America, USA) to measure the pH value, PaO2 and PaCO2.

### Lung wet/dry weight ratio

The lower lobe of the right lung was removed, the surface was rinsed, and then the wet weight was measured. The lung tissue was then dried at 80 °C in an oven for 48 h and reweighed to obtain the dry weight (the difference between the two measurements was less than 0.05 mg). The lung tissue W/D ratios were calculated.

### Western blotting analysis

Total protein was extracted from lung tissue and RPMVECs. A total of 10 μg protein per lane was resolved by 8–15% SDS‒PAGE and transferred using electroblotting to PVDF membranes. The membranes were blocked with 5% nonfat milk and incubated with primary antibodies against cleaved caspase3 (1:1000 dilution), Bcl-2 (1:1000 dilution), Bax (1:1000 dilution) and β-actin (1:1000 dilution) overnight. Next, the membranes were washed with PBS solution three times, incubated with HRP-conjugated anti-rabbit or anti-mouse secondary antibodies (1:4000 dilution) at room temperature for 1 h and developed using an enhanced chemiluminescence detection kit (EMD Millipore). The band intensity was analyzed using ImageJ software (National Institutes of Health, Bethesda, MA, USA). β-actin was used for normalization (Representative images for each western blot result presented in Supplementary information).

### Ethics statement

The study was carried out in compliance with the ARRIVE guidelines. The animal experiments involved in this study have been approved by the Ethics Committee of the Huazhong University of Science and Technology. All experiments were performed in accordance with relevant guidelines and regulations.

### Statistical analysis

All results are expressed as the mean ± standard deviation. All data were obtained by repeated three times. The data were analyzed by using SPSS 20.0 (IBM, Chicago, USA) and GraphPad Prism 5.0 Statistical differences between multiple groups were determined using one-way ANOVA followed by Tukey’s post hoc test. Comparisons between two groups were analyzed using the unpaired Student’s t-test. P < 0.05 was considered to indicate a statistically significant difference.

### Supplementary Information

Supplementary Figure 2.

Supplementary Figure 3.

Supplementary Figure 4.

Supplementary Figure 5.

Supplementary Figure 6.

Supplementary Figure 7.

Supplementary Figure 8.

Supplementary Figure 9.

Supplementary Figure 10.

Supplementary Figure 11.

Supplementary Figure 12.

Supplementary Figure 13.

Supplementary Figure 14.

Supplementary Figure 15.

Supplementary Figure 16.

## Data Availability

The datasets analyzed during the current study are available from the corresponding author on reasonable request.
