# Supplementary figures and images for: Protective effects of butorphanol in oleic acid-endotoxin “two-hit” induced rat lung injury by suppression of inflammation and apoptosis
Source: Sci Rep. 2024 Jun 20;14:14231. doi: 10.1038/s41598-024-53483-5 (PMC11190203; doi:10.1038/s41598-024-53483-5)

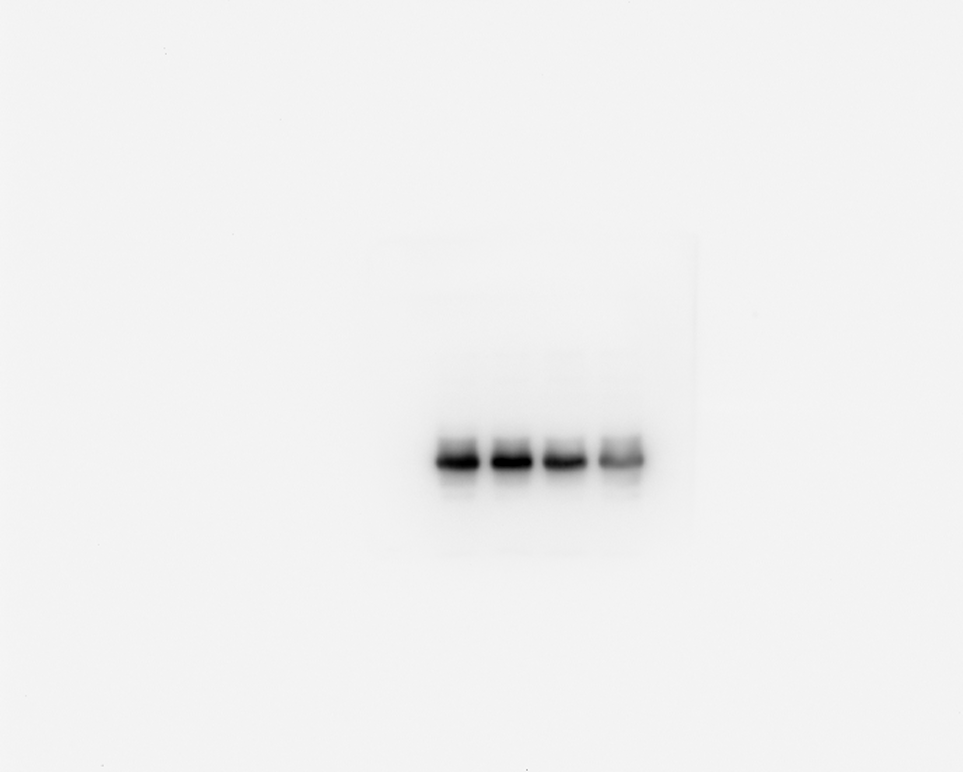

Supplement: Supplementary file 1 — Supplementary Figure 2. [file 41598_2024_53483_MOESM1_ESM.tif]

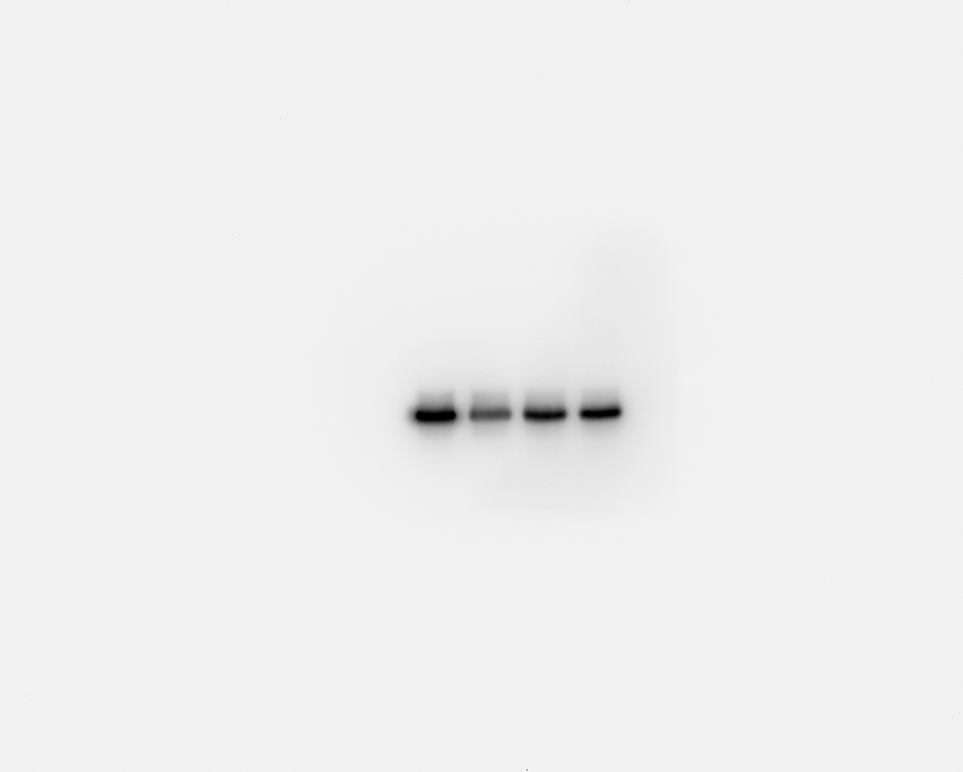

Supplement: Supplementary file 2 — Supplementary Figure 3. [file 41598_2024_53483_MOESM2_ESM.tif]

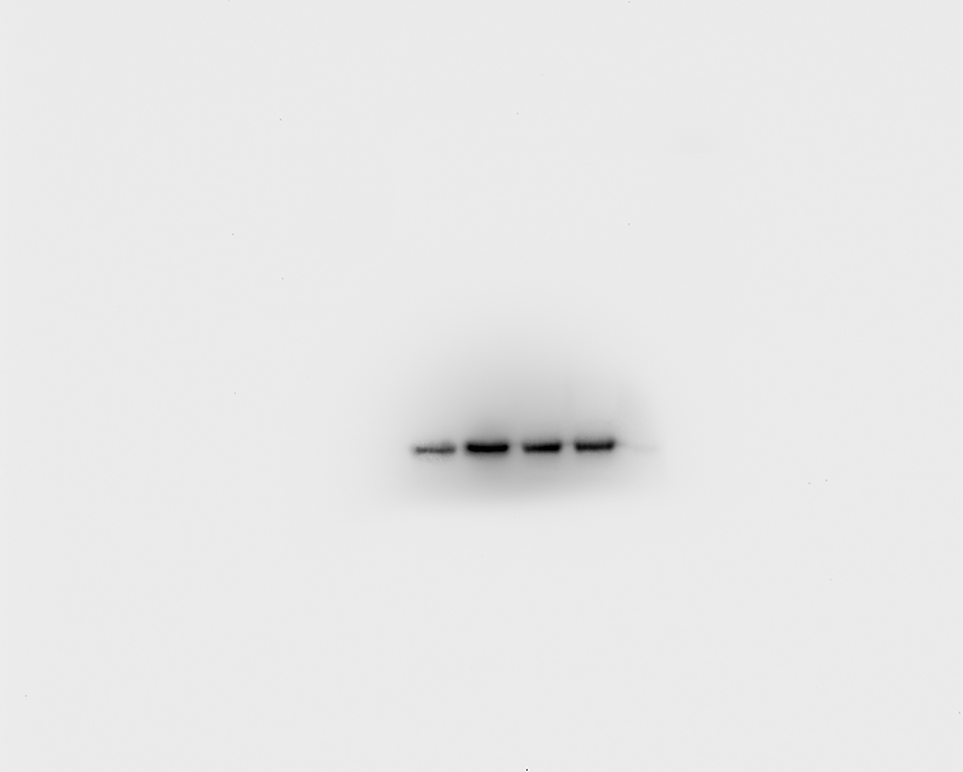

Supplement: Supplementary file 3 — Supplementary Figure 4. [file 41598_2024_53483_MOESM3_ESM.tif]

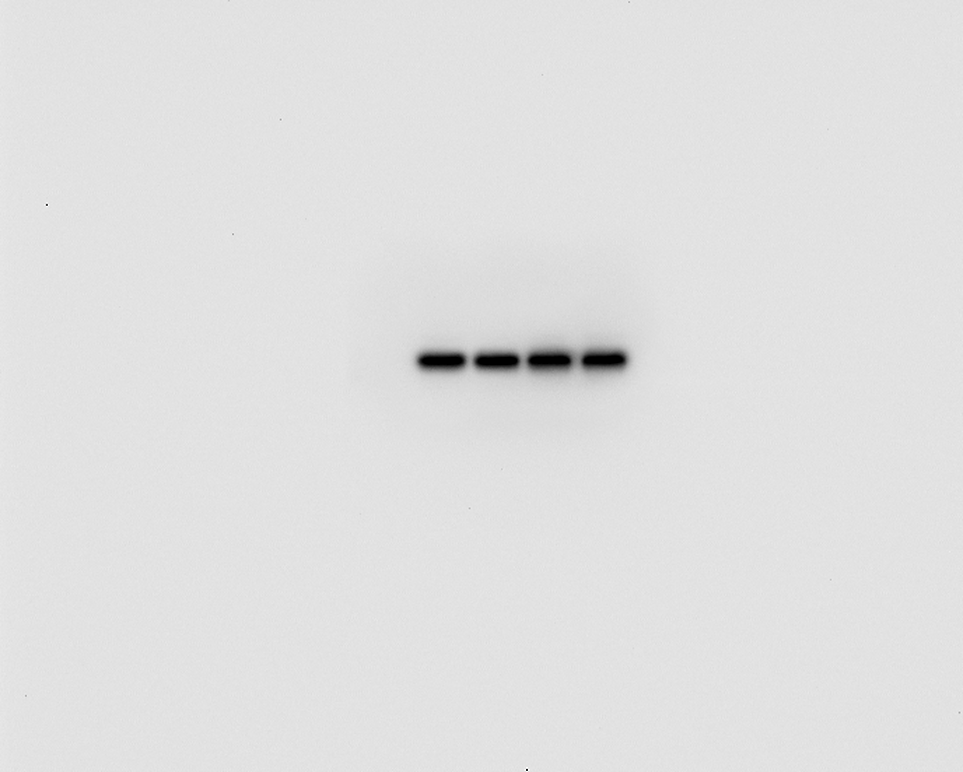

Supplement: Supplementary file 4 — Supplementary Figure 5. [file 41598_2024_53483_MOESM4_ESM.tif]

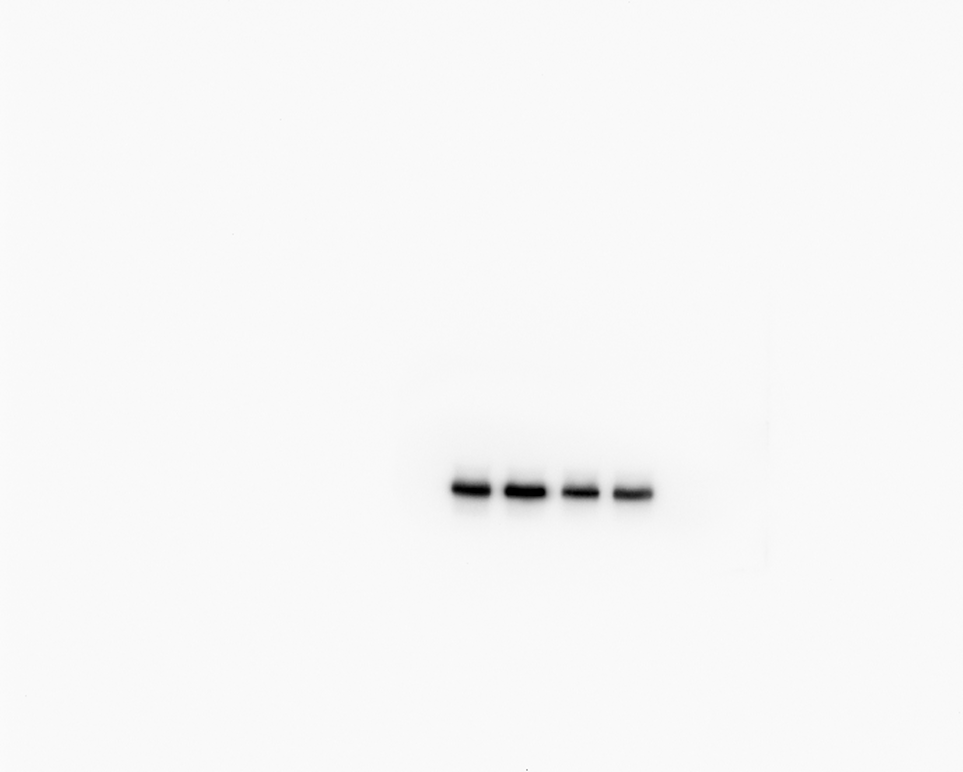

Supplement: Supplementary file 5 — Supplementary Figure 6. [file 41598_2024_53483_MOESM5_ESM.tif]

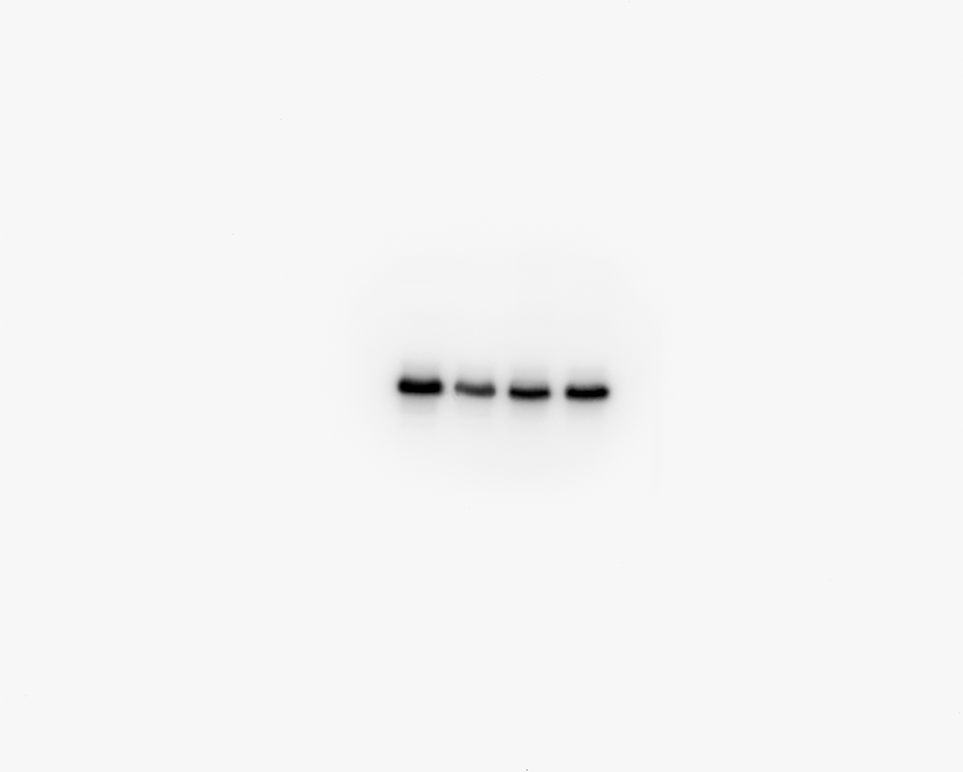

Supplement: Supplementary file 6 — Supplementary Figure 7. [file 41598_2024_53483_MOESM6_ESM.tif]

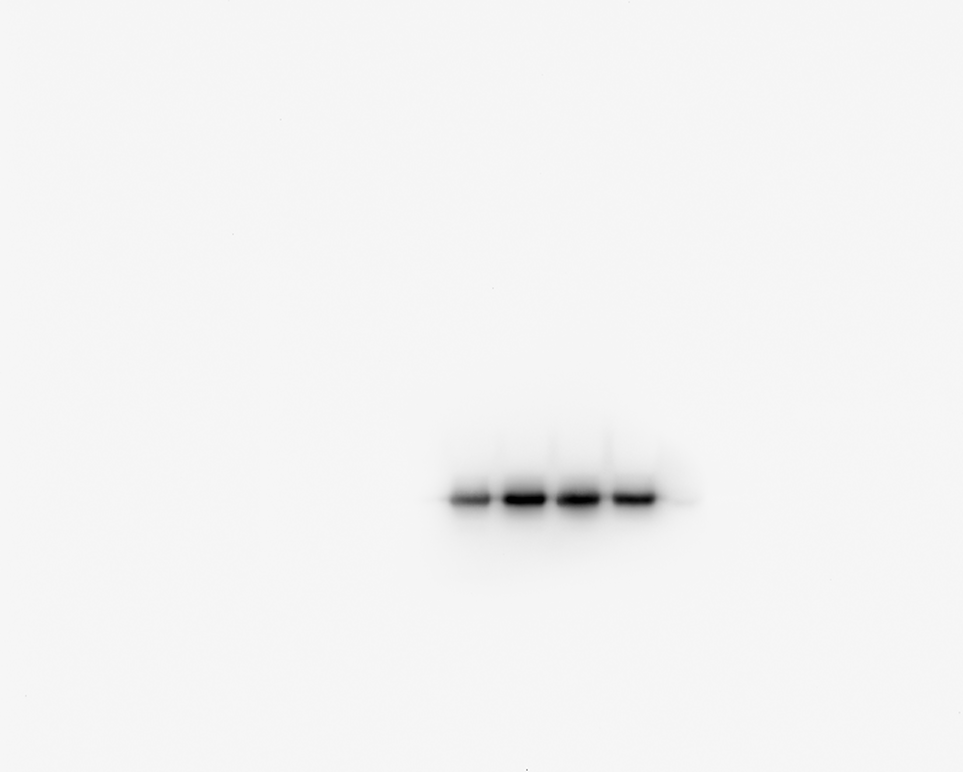

Supplement: Supplementary file 7 — Supplementary Figure 8. [file 41598_2024_53483_MOESM7_ESM.tif]

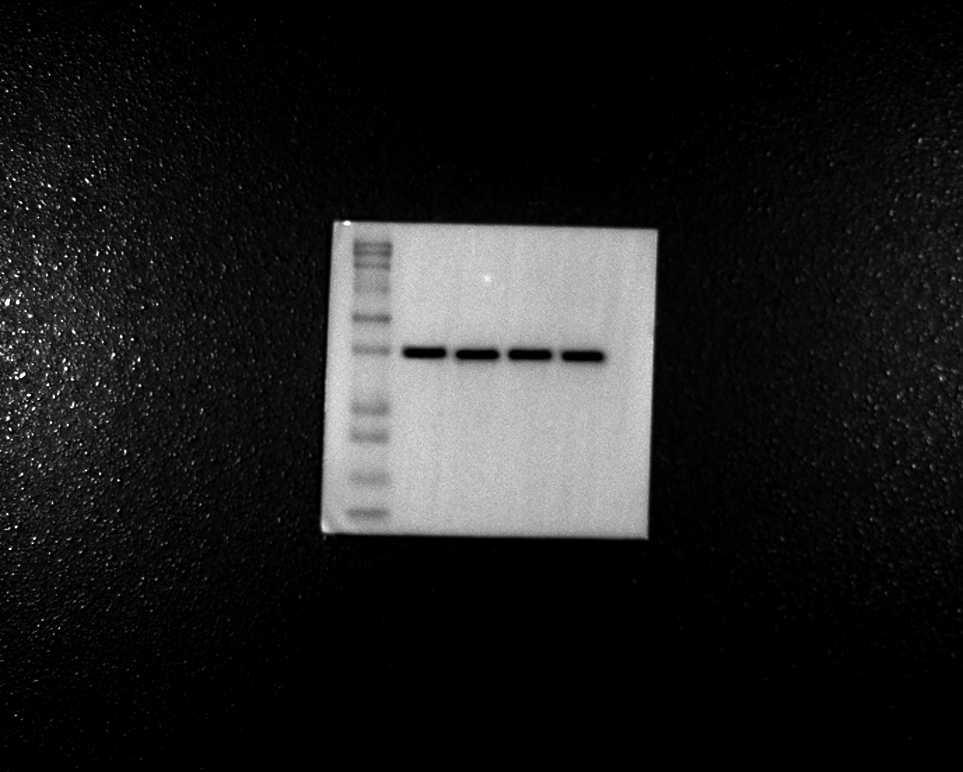

Supplement: Supplementary file 8 — Supplementary Figure 9. [file 41598_2024_53483_MOESM8_ESM.tif]

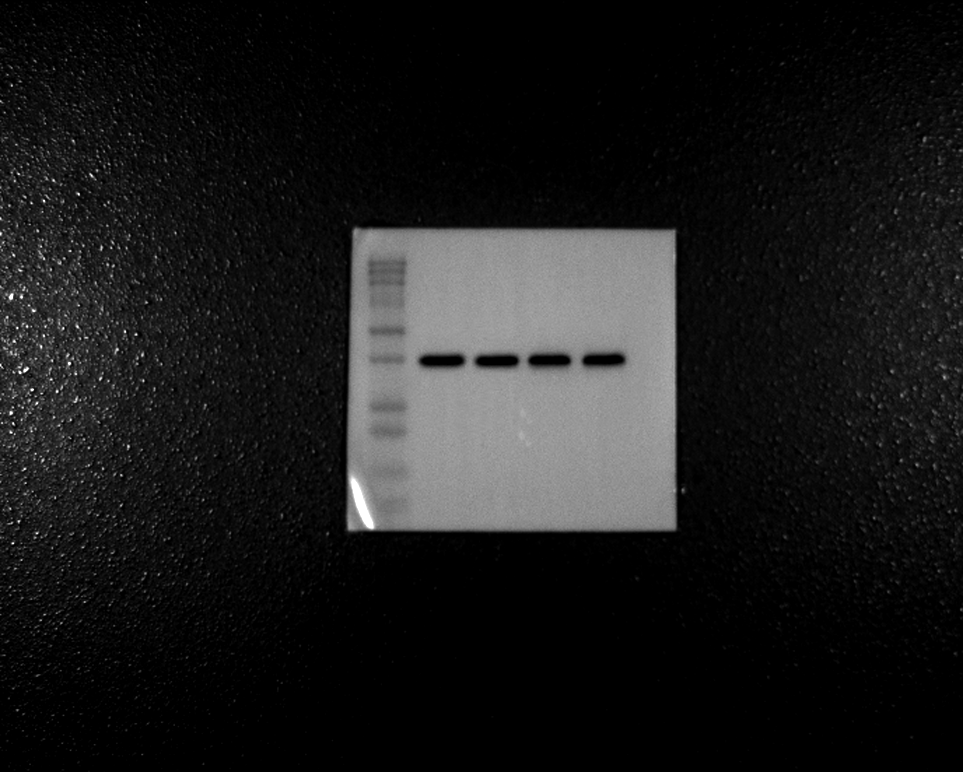

Supplement: Supplementary file 9 — Supplementary Figure 10. [file 41598_2024_53483_MOESM9_ESM.tif]

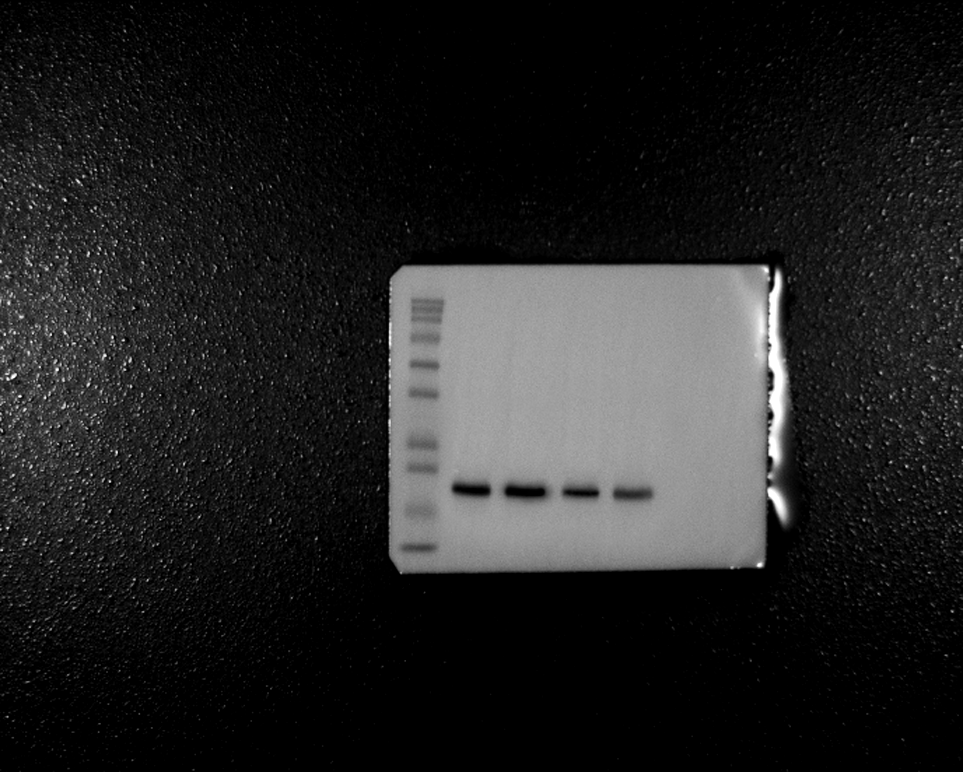

Supplement: Supplementary file 10 — Supplementary Figure 11. [file 41598_2024_53483_MOESM10_ESM.tif]

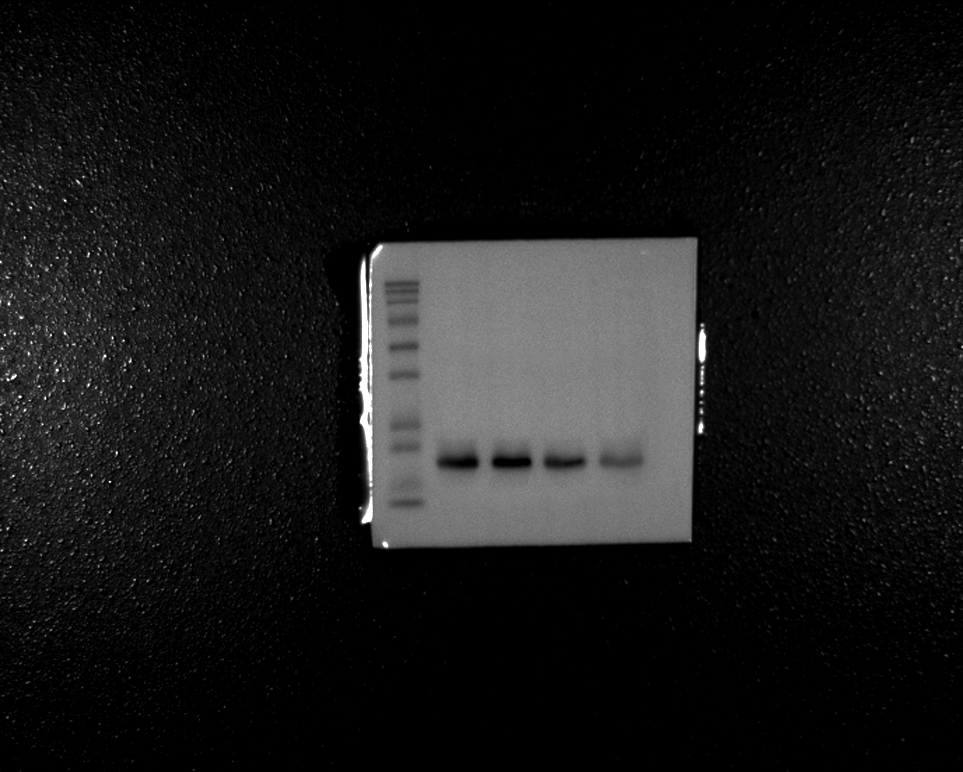

Supplement: Supplementary file 11 — Supplementary Figure 12. [file 41598_2024_53483_MOESM11_ESM.tif]

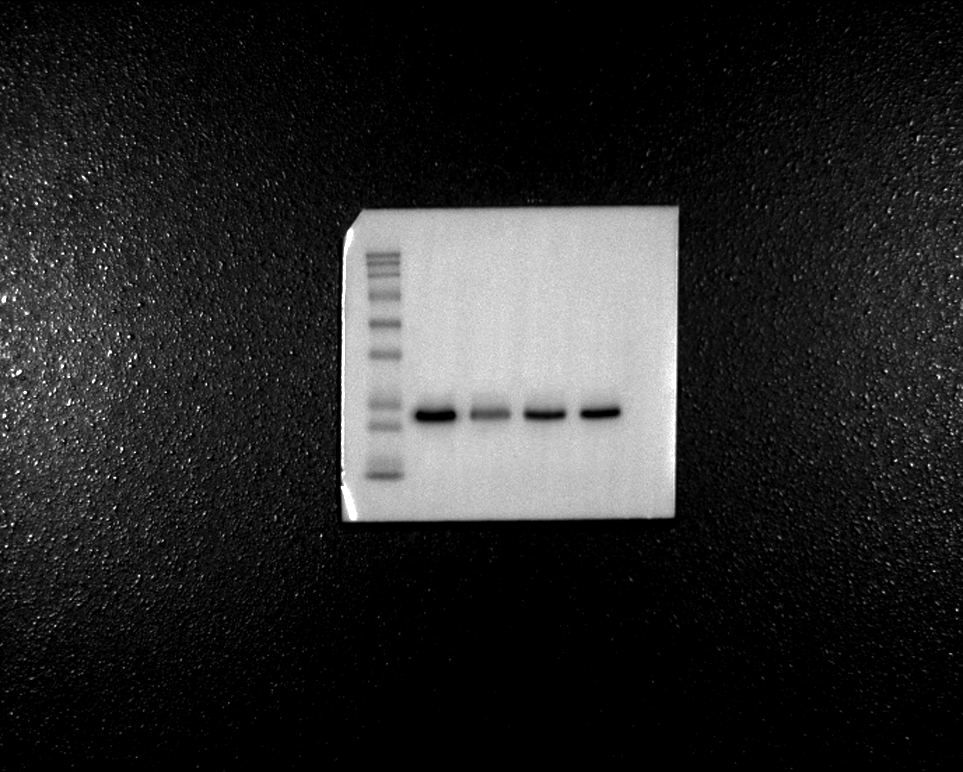

Supplement: Supplementary file 12 — Supplementary Figure 13. [file 41598_2024_53483_MOESM12_ESM.tif]

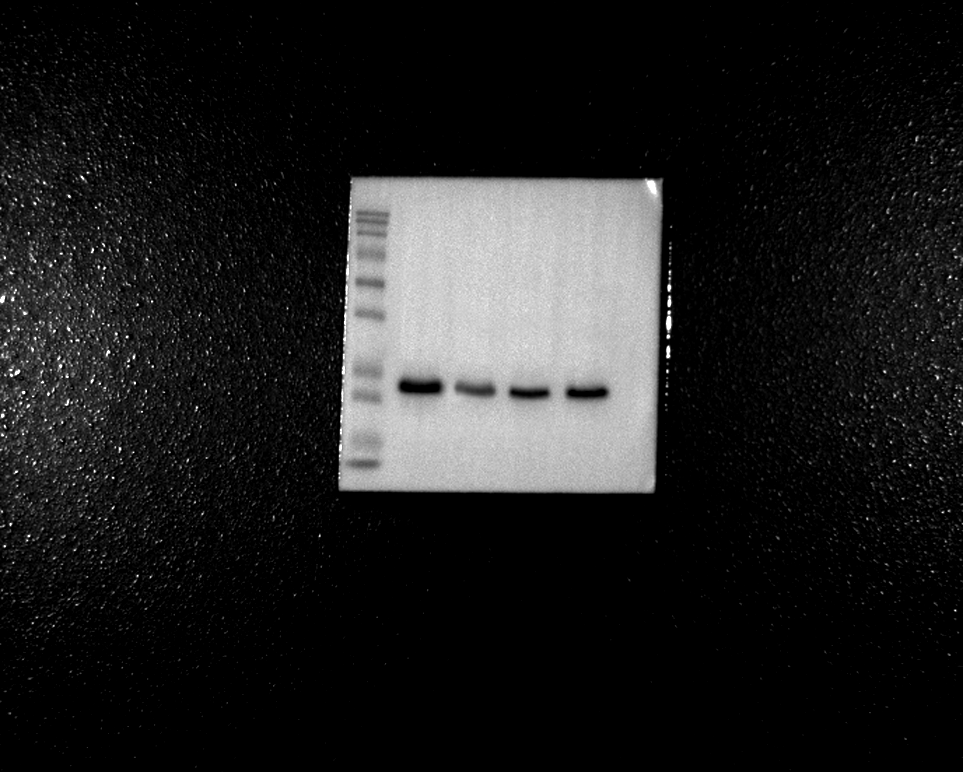

Supplement: Supplementary file 13 — Supplementary Figure 14. [file 41598_2024_53483_MOESM13_ESM.tif]

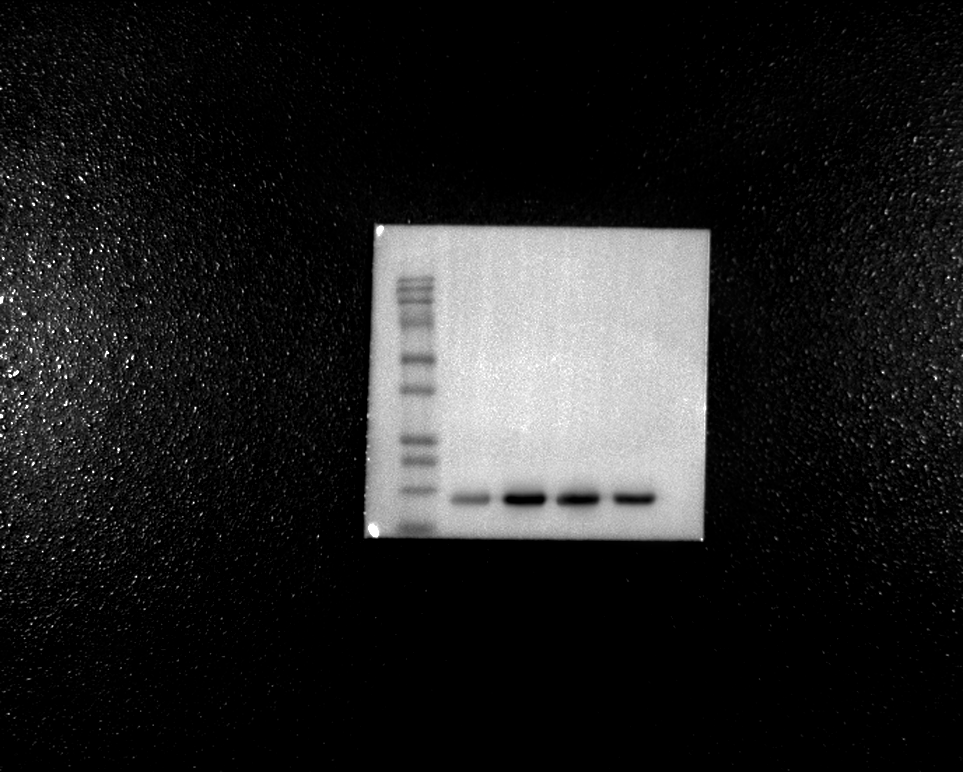

Supplement: Supplementary file 14 — Supplementary Figure 15. [file 41598_2024_53483_MOESM14_ESM.tif]

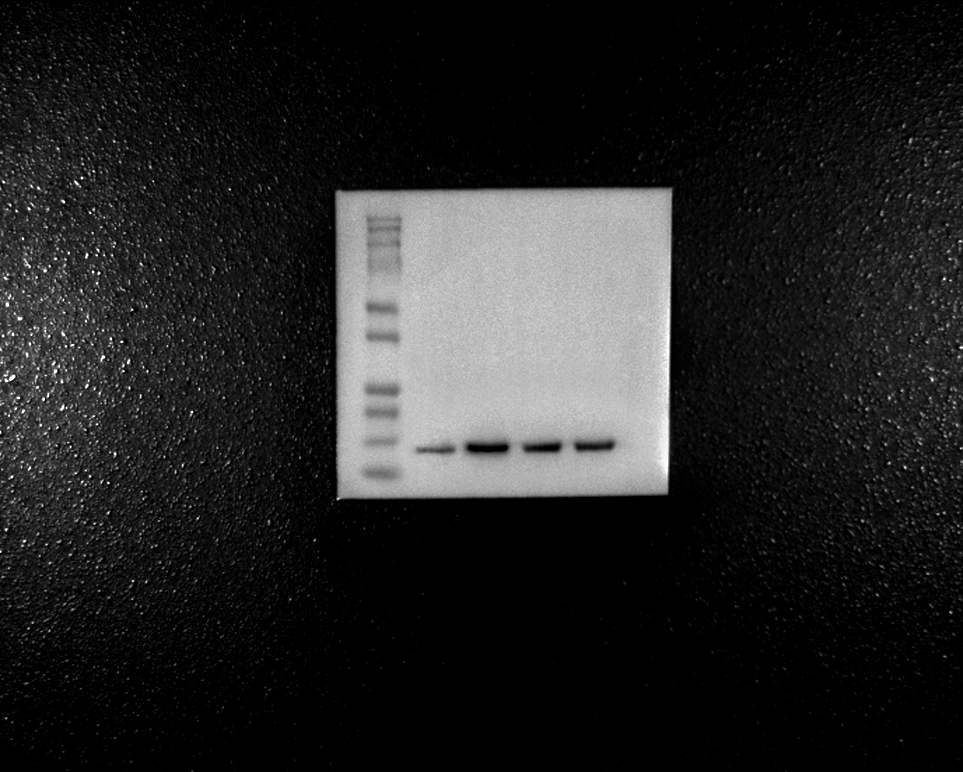

Supplement: Supplementary file 15 — Supplementary Figure 16. [file 41598_2024_53483_MOESM15_ESM.tif]
